# Draft Genome Sequences of Clinical and Environmental Isolates of Aspergillus tamarii from Colombia

**DOI:** 10.1128/MRA.01514-19

**Published:** 2020-04-02

**Authors:** Oscar M. Gómez, Carmen G. Freyle, Susana Torres, Álvaro L. Rúa, Diana P. Tamayo, Juan G. McEwen, Clayton L. Borges, Orville Hernández

**Affiliations:** a Cellular and Molecular Biology Unit, Corporación para Investigaciones Biológicas (CIB), Medellín, Colombia; b School of Microbiology, Universidad de Antioquia, Medellín, Colombia; c MICROBA Research Group, School of Microbiology, Universidad de Antioquia, Medellín, Colombia; d Microbiología Ambiental Group, School of Microbiology, Universidad de Antioquia, Medellín, Colombia; e School of Medicine, Universidad de Antioquia, Medellín, Colombia; f Laboratório de Biologia Molecular, Universidade Federal de Goiás, Goiás, Brazil; Broad Institute

## Abstract

*Aspergillus* is a very diverse genus of fungi that are common in the environment and can affect human health. Here, we report the draft genome sequences of two Colombian isolates of Aspergillus tamarii, an emerging pathogenic species. One isolate was obtained from an infected patient and the other from the environment in a hospital.

## ANNOUNCEMENT

The genus *Aspergillus* is a group of opportunistic fungi that cause infections with high morbimortality in immunocompromised hosts (1). Approximately 350 species have been described in this genus, classified into 7 subgenera and 22 sections (2, 3). In Colombia, A. fumigatus is one of the most frequently isolated species causing infection (4). In recent years, the isolation of other *Aspergillus* species in hospital environments has been reported; however, the relationship between environmental and clinical isolates had not been established (5). Aspergillus tamarii is an emerging pathogenic species of the section *Flavi*, which has been associated with a wide spectrum of clinical manifestations (6). Thus, the aim of this work was to characterize the genotypes of *Aspergillus tamarii* isolates obtained from a Colombian hospital (clinical and environmental sources).

First, we collected two Colombian samples of *Aspergillus tamarii*, one from a patient with an infected wound and the other from the indoor environment of the same hospital. The isolates were identified according to their macroscopic and microscopic characteristics in peptone-dextrose agar (PDA), malt extract agar (MEA), and Czapek yeast autolysate (CYA) agar culture media, as described by Samson and coworkers (2). The isolates were cultured in brain heart infusion (BHI) medium supplemented with 1% glucose at 20°C and 120 rpm. The biomass was collected during the exponential growth phase after 96 h of incubation. Genomic DNA for sequencing was prepared from mycelium culture using phenol-chloroform extraction (7). Approximately 1 μg of DNA (optical density at 260/289 nm [OD_260/289_] ratio, 1.8 to 2.0) was used to prepare 170- to 800-bp libraries, and 150-bp paired-end sequencing was performed using the Illumina HiSeq 4000 platform. A total of 13,498,096 raw reads were generated for strain UdeA_Ata2 and 13,602,390 for strain UdeA_Afl2. The low-quality reads (1.17 and 1.3%, respectively) and adapter sequences were removed after FastQC v0.11.5 analysis with default settings (8). The high-quality (Q score, >30) reads were assembled *de novo* using the SPAdes v3.10 pipeline with the BayesHammer module for error correction, iterative k-mer lengths (21, 33, 55, and 77 bp), and the “careful” option (9). Scaffolds smaller than 500 bp were filtered out. The draft genome assembly quality was analyzed with QUAST v4.5 using default settings (10). The assembled scaffolds generated by the isolates were aligned and oriented with Aspergillus flavus NRRL3357 (GenBank accession number GCF_000006275.2) using MAUVE v2.4.0 (10). AUGUSTUS v3.0.1 was used for gene prediction based on gene models from Aspergillus oryzae (11). The assembly statistics are shown in Table 1.

**TABLE 1:** Summary of assembly statistics

Sample	Species	Source	Genome size (Mb)	No. of reads	No. of scaffolds	Scaffold *N*_50_ (bp)	Largest scaffold (Mb)	Coverage (×)	No. of genes	G+C content (%)	Accession no.
UdeA_Ata2	*Aspergillus tamarii*	Hospital environment (air)	38.2	13,498,096	911	101,804	0.53	32	13,771	47.62	VYTV000000000
UdeA_Afl2	*Aspergillus tamarii*	Left foot biopsy	37.7	13,602,390	593	145,682	0.80	33	13,630	47.63	VYTW00000000

**FIG 1:**
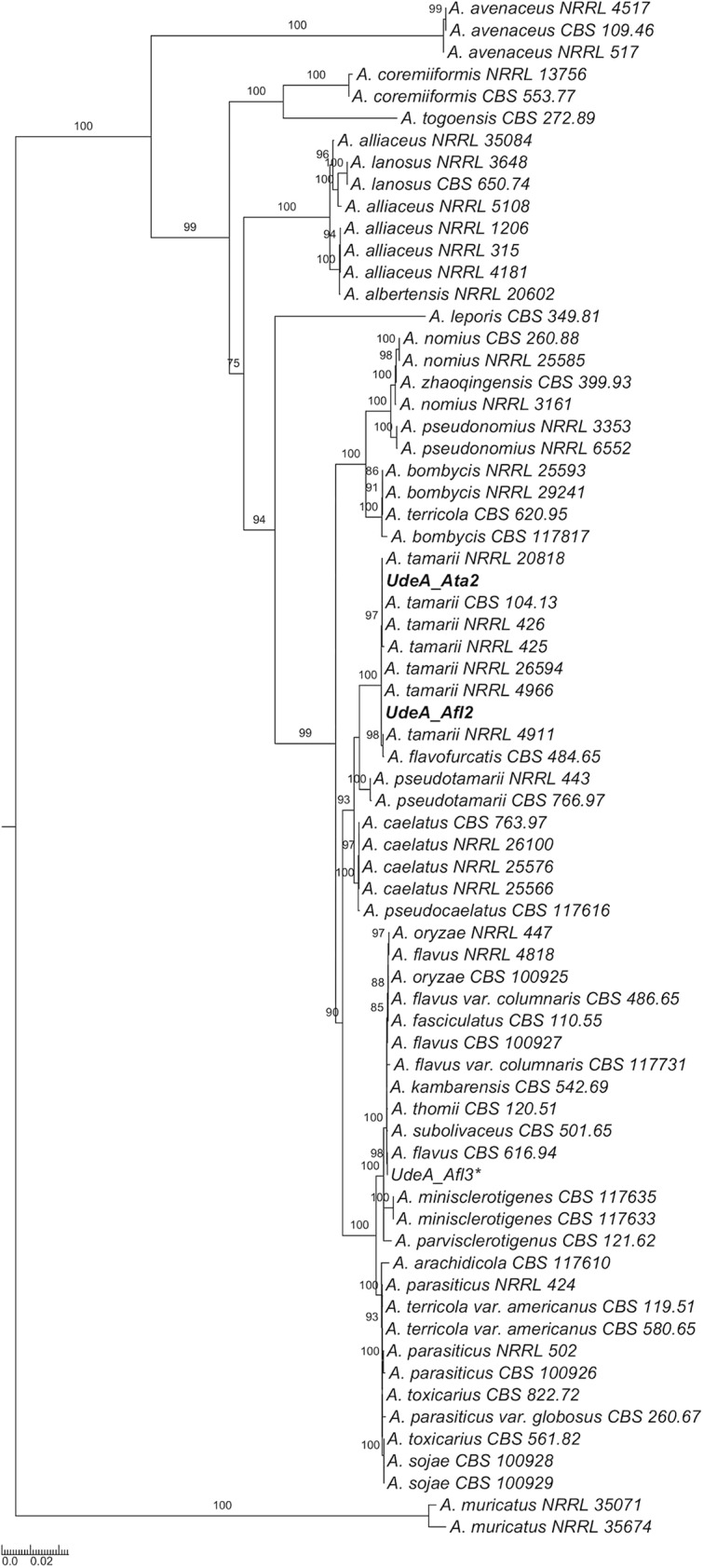
Phylogenetic reconstruction of the *Aspergillus* section *Flavi* using the maximum likelihood (ML) method with IQ-TREE software based on concatenated sequences of the markers ITS, *cmdA*, and *benA*. The numbers near each branch show indices of support that are equal to or greater than 70 based on 1,000 ultrafast bootstrap replications. The Colombian *A. tamarii* isolate is shown in bold. Aspergillus muricatus (section *Circumdati*) was used as an outgroup.*, unpublished data.

In order to classify the species of the *Aspergillus* isolates by barcoding, we identified the sequences of the internal transcribed spacer (ITS), *cmdA*, and *benA* markers in the assemblies using BLAST v2.2.25 with default settings (12); only one copy of these genes was found in each of the two assemblies. These sequences were aligned against RefSeq entries for *Aspergillus* spp. from DDBJ/EMBL/GenBank (https://doi.org/10.6084/m9.figshare.11862822) using MAFFT (https://www.ebi.ac.uk/Tools/msa/mafft/). The retrieved alignments were manually checked and concatenated. IQ-TREE v1.4.4 software was used for phylogenetic reconstruction with the maximum likelihood (ML) method with the options “-m TEST” and “–sp partitioned matrix” (13). TreeGraph 2 v1.4.4 was used for tree visualization (14). In the phylogeny, the two isolates sequenced were grouped with reference strains of *Aspergillus tamarii* (Fig. 1).

### Data availability.

These whole-genome sequences were deposited at DDBJ/ENA/GenBank under the accession numbers VYTV000000000 and VYTW00000000. The raw sequence reads have been deposited in the NCBI Sequence Read Archive under BioProject number PRJNA529233. The aggregated data are available on Figshare at https://doi.org/10.6084/m9.figshare.11862648.
